# Usefulness of readout-segmented EPI-based diffusion tensor imaging of lacrimal gland for detection and disease staging in thyroid-associated ophthalmopathy

**DOI:** 10.1186/s12886-021-02044-9

**Published:** 2021-07-20

**Authors:** Lu Chen, Hao Hu, Wen Chen, Qian Wu, Jiang Zhou, Huan-Huan Chen, Xiao-Quan Xu, Hai-Bin Shi, Fei-Yun Wu

**Affiliations:** 1 Department of Radiology, The First Affiliated Hospital of Nanjing Medical University, Address: No. 300, Guangzhou Rd, Gulou district, Nanjing, P.R. China; 2 Department of Endocrinology, The First Affiliated Hospital of Nanjing Medical University, Nanjing, China

**Keywords:** Thyroid-associated ophthalmopathy, Lacrimal gland, Diffusion tensor imaging, Readout-segmented echo-planar imaging

## Abstract

**Background:**

Dysfunction of lacrimal gland (LG) gains increasing attention in patients with thyroid-associated ophthalmopathy (TAO), while the underlying pathological change is still not fully established. This study aimed to evaluate the utility of readout-segmented echo-planar imaging (rs-EPI)-based diffusion tensor imaging (DTI) in non-invasively detecting microstructural alterations of LG in patients with TAO, as well as in discriminating disease activity.

**Methods:**

Thirty TAO patients and 15 age- and sex- matched healthy controls, who underwent rs-EPI-based DTI, were retrospectively enrolled. Fractional anisotropy (FA) and apparent diffusion coefficient (ADC) of LG, and clinical-endocrinological variables were collected and compared. The correlations between FA and ADC values of LG and serum thyroid biochemical markers were also assessed.

**Results:**

TAO group showed significantly lower FA (*P* < 0.001) and higher ADC (*P* = 0.014) of LG than healthy group. Active subgroup had significantly lower FA (*P* < 0.001) and higher ADC (*P* < 0.001) than inactive subgroup. In TAO group, FA of LG was significantly and negatively correlated with TRAb (*r*=-0.475, *P* = 0.008), while ADC of LG showed no significant correlation (*P* > 0.05). The area under receiver operating characteristic curve of FA was significantly greater than that under curve of ADC for discriminating disease activity (0.832 vs. 0.570, *P* = 0.009).

**Conclusions:**

rs-EPI-based DTI is a useful tool to characterize the microstructural change of LG in patients with TAO. The derived metrics, particularly FA, can help to reveal disease activity.

## Background

Thyroid-associated ophthalmopathy (TAO), also known as Graves’ ophthalmopathy, is the most common extrathyroidal manifestation of Graves’ disease [1, 2]. Patients with TAO commonly complain about biliousness of ocular surface, including photophobia, excess tearing, grittiness, and foreign-body sensations [3, 4]. Besides the traditionally documented pathogeneses regarding anatomical changes of widened vertical palpebral fissure and increased exophthalmos that accelerate the evaporation of tears, decreased secretion of tears due to lacrimal gland (LG) involvement has been considered as another crucial factor [3–6]. A strong correlation between thyroid antibodies and lacrimal dysfunction has also been declared, indicating the target role of LG in disease process of TAO [3, 7].

Recently, along with the development of imaging techniques, a few studies have investigated the dysfunction of LG in TAO cohort through non-invasive approaches [6, 8–10]. Computed tomography imaging-based morphologic measurements of LG have been demonstrated to be useful in the diagnosis of TAO [8, 9], while low soft tissue resolution and inevitable radiation exposure limited its wide utilization. Signal intensity ratio and herniation of LG derived from magnetic resonance imaging (MRI) have been reported to be useful in grading disease activity [6, 10]. However, the change of LG was still assessed in morphological and semi-quantitative approaches, underlying pathological information of LG remains not fully studied.

Diffusion tensor imaging (DTI), as an advanced MRI technique, can provide quantitative information about the microstructural integrities of oriented tissues in three-dimensional space [11, 12]. The extracted apparent diffusion coefficient (ADC) and fractional anisotropy (FA) values can reflect the magnitude and directionality of water diffusion, respectively [11, 12]. Beyond the initial application in fiber bundles like nerves and muscles [13, 14], the metrics have been increasingly extended to assess the microstructural architecture of various normal and diseased tissues, including breast, prostate, liver, kidney and so on [15–17]. In head and neck domain, besides the previous applications on muscles of tongue, orbit and pterygoid [18, 19], recent studies also showed the usefulness of DTI in diagnosing salivary gland tumors [20, 21]. However, the data about the ability of DTI to detect the microstructural changes of LG are still lacked.

Moreover, single-shot echo-planar imaging (ss-EPI) sequence was usually employed to acquire DTI in previous studies [13, 14, 18–21]. However, the frequent susceptibility artifact, especially in poor magnetic field homogeneity and high field strength, may raise the difficulty for accurate assessment [22]. As a potentially modified approach, readout-segmented EPI (rs-EPI) has received more and more attention by dividing k-space into several segments along the readout direction [23]. It has been verified to assist in improving image quality accompanied with superior normal anatomical structure distinction than ss-EPI [22, 23].

Therefore, the purpose of this study was to evaluate the utility of rs-EPI-based DTI in detecting microstructural changes of LG in TAO patients, as well as in discriminating disease activity, since treatment strategies differed significantly between patients with active and inactive processes.

## Methods

### Patients

This study followed the tenets of the declaration of Helsinki and was approved by the Institutional Review Board of the First Affiliated Hospital of Nanjing Medical University. The informed consent requirement was waived due to its retrospective nature. All methods were carried out in accordance with relevant guidelines and regulations. We enrolled 30 consecutive patients (mean age, 46.4 ± 13.4 years; male/female ratio, 12/18) who were clinically diagnosed with TAO from May 2017 to March 2018. The exclusion criteria were as follows: (1) rs-EPI-based DTI was not acquired during pre-treatment orbital MRI; (2) image quality was inadequate for further analysis; (3) history of radiotherapy, surgical rehabilitation or decompression; (4) other ophthalmopathies such as glaucoma, diabetic retinopathy, and uveitis; (5) other immunological disorders such as IgG4-related disease and idiopathic inflammatory pseudotumor.

Disease activity was evaluated using the modified 7-item clinical activity score (CAS) and determined for each eye [24]. If CAS ≥ 3, the eye was classified into active phase, otherwise inactive (CAS < 3). Totally, 30 active and 30 inactive eyes were defined. Associated clinical-endocrinological variables: age, sex, diplopia presence, and serum thyroid biochemical levels of free triiodothyronine (FT3), free thyroxine (FT4), thyroid-stimulating hormone (TSH), and thyrotrophin receptor antibody (TRAb), were also collected. In addition, 15 age- and sex-matched healthy controls (HCs) were enrolled during the same period and underwent the same MRI protocol. All the HCs were in hematologically euthyroid status and had no symptoms caused by ophthalmopathies and immunological disorders.

### Image acquisition

A 3.0-T MRI scanner (Magnetom Skyra; Siemens Healthcare, Erlangen, Germany) equipped with a 20-channel head coil was used. Patients were asked to rest in supine position and look at a fixed site with eyes closed in order to reduce motion-related artifacts. Orbital DTI was performed on an axial plane by using rs-EPI sequence. Detailed imaging parameters include: repetition time/ echo time = 2000/83 ms; readout segments = 5; field of view = 220 × 220 mm^2^; matrix = 190 × 171; slice thickness = 3mm; b = 0, 1000 s/mm^2^; non-collinear gradient encoding directions = 30. Conventional protocols included axial T1-weighted imaging (repetition time/ echo time = 635/6.7 ms), axial, coronal, and sagittal T2-weighted imaging with fat suppression (repetition time/ echo time = 4000/75–117 ms).

### Image analysis

**Fig. 1:**
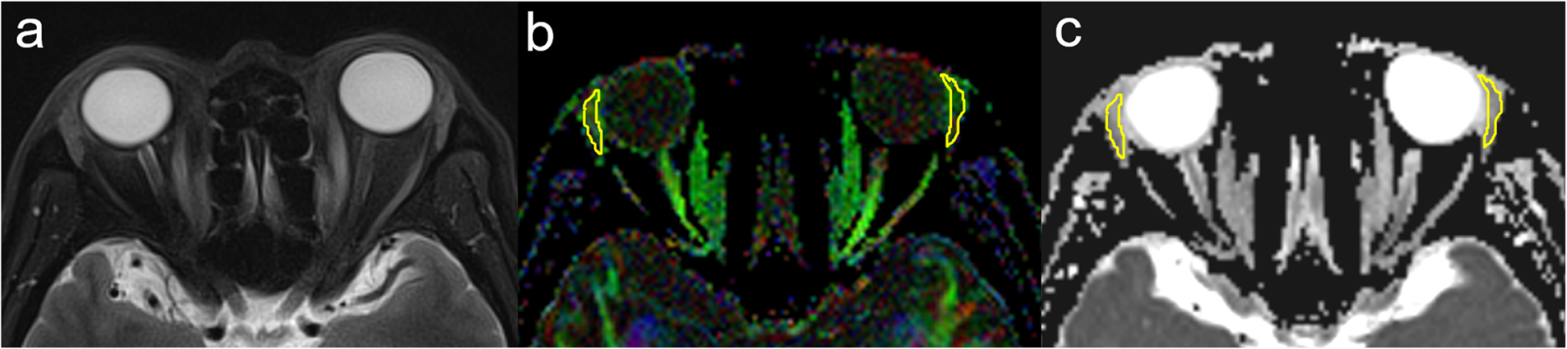
Schematic diagrams for measurements of LG on rs-EPI-based DTI. Figure **a** showed the axial fat-suppressed T2-weighted imaging in a 54-year old male with TAO. Figure **b** and **c** showed the corresponding axial color-coded FA and ADC maps, respectively. A polygonal region of interest was manually set on each LG locating the maximum cross-section, with careful avoidance of surrounding tissues. LG: lacrimal gland, rs-EPI-based DTI: readout-segmented echo-planar imaging-based diffusion tensor imaging, TAO: thyroid-associated ophthalmopathy, FA: fractional anisotropy, ADC: apparent diffusion coefficient

Postprocessing of rs-EPI-based DTI was performed by using the standalone platform (Syngo Via, Siemens Healthcare, Erlangen, Germany). FA and ADC maps were calculated on a pixel-by-pixel basis. A polygonal region of interest was manually set on each LG locating the maximum cross-section on axial rs-EPI-based DTI, with careful avoidance of surrounding tissues (Fig. 1). The mean FA and ADC values over the region of interest were collected.

Two experienced neuroradiologists who were blinded to the clinical conditions of the subjects independently processed the data. The measurement results of these two observers were used to assess the inter-observer agreement. All the measurements were repeated by observer 1 with a washout period of at least 1 month, for evaluating the intra-observer reproducibility.

### Statistical analysis

Numeric data were reported as mean ± standard deviation. Kolmogorov-Smirnov test was used for normality distribution analysis. A two-factor split-plot ANOVA for ‘within subject’ correlation correction [25] was applied to compare mean group values of FA and ADC of LG. Independent-sample t-test was used to compare the difference on age between groups. Chi-square test was applied to compare the differences of sex and diplopia presence between groups. Mann-Whitney U test was used to compare mean group values of FT3, FT4, TSH, TRAb, and CAS. Spearman’s rank correlation coefficients between FA and ADC of LG and serum thyroid biochemical markers were calculated. Receiver operating characteristic curve analysis was performed to evaluate the ability of FA and ADC to discriminate disease activity.

The inter- and intra-observer reproducibility of the measurements were assessed by using intraclass correlation coefficients (ICCs) with 95 % confidence intervals. A two-way ICC with random rater assumption was used. ICCs < 0.40 represent poor reproducibility, 0.40–0.60 as moderate, 0.61–0.80 as good, and ≥ 0.81 as excellent [26]. Two-sided *P* values of < 0.05 were considered significant. All statistical analyses were conducted by the SPSS software (v. 23.0; IBM, Armonk, NY).

## Results

Clinical and demographic data of 30 TAO patients and 15 HCs were summarized in Table 1. There were no significant differences on age and sex distribution between TAO and HC groups (*P* > 0.05), as well as between active and inactive subgroups (*P* > 0.05). There were also no significant differences on thyroid biochemical levels of FT3, FT4, TSH and diplopia presence between active and inactive subgroups (*P* > 0.05). Serum level of TRAb differed significantly between active and inactive patients (4.50 ± 3.49 vs. 3.91 ± 8.09, *P* = 0.005). The mean CAS of active patients was significantly higher than that of inactive ones (4.6 ± 0.8 vs. 1.3 ± 0.8, *P* < 0.001).

**Table. 1:** Clinical and demographic information of study population

	**TAO (*N*=30)**			**HC **	^***a***^***P ***
**Variables**	**Active(*N*=15)**	**Inactive(*N*=15)**	^***b***^***P***	**(*N*=15)**	
**Age (year)**	46.4±13.4			46.5±13.5	0.981
	47.8±9.9	44.9±16.4	0.566		
**Sex (M/F)**	12/18			6/9	1.000
	7/8	5/10	0.456		
**FT3 (pmol/L)**	6.76±7.43	6.38±3.60	0.300		
**FT4 (pmol/L)**	20.03±16.96	16.66±6.25	0.950		
**TSH (mIU/L)**	1.230±1.872	2.018±2.204	0.530		
**TRAb (IU/L)**	4.50±3.49	3.91±8.09	0.005		
**Diplopia presence**	8	6	0.464		
**CAS**	4.6±0.8	1.3±0.8	<0.001		

^a^*P* Comparisons between TAO and HC

^b^*P* Comparisons between active phase and inactive phase

*TAO* thyroid-associated ophthalmopathy, *HC* healthy control, *M* male, *F* female, *CAS* clinical activity score

**Fig. 2:**
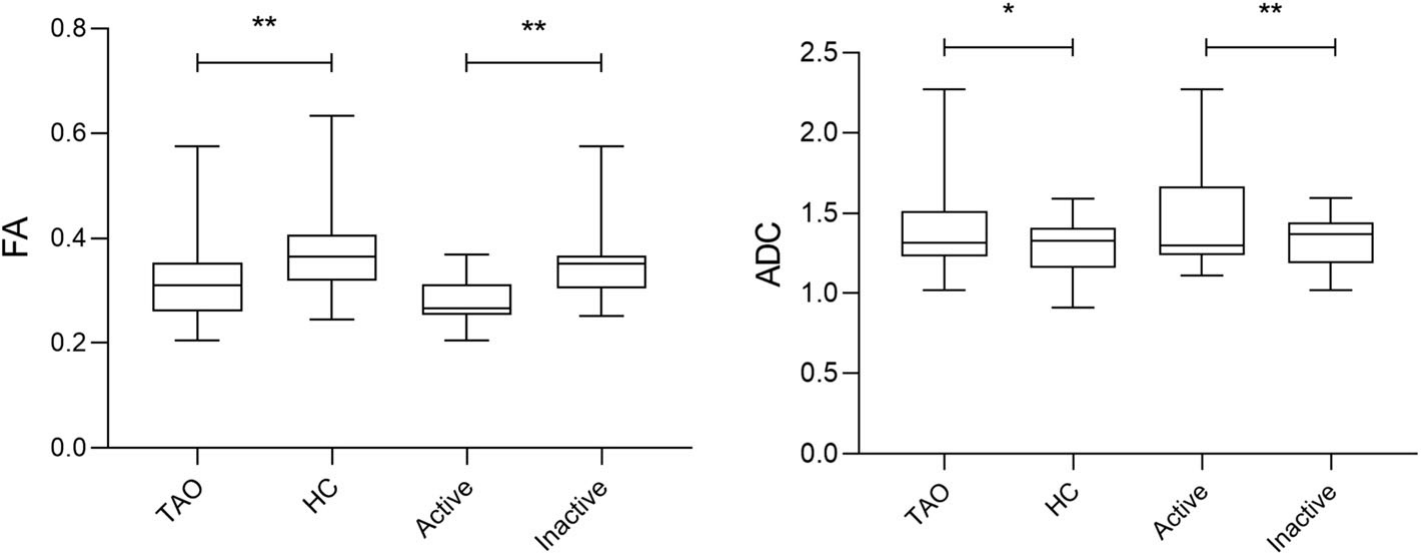
Box-and-whisker plots showing the comparisons of FA and ADC of LG between groups. The unit of ADC is ×10^− 3^ mm^2^/s. The asterisk indicates a significant difference (^**^P < 0.001, ^*^P < 0.05)

Excellent intra- and inter-observer reproducibility were obtained for measurements of all rs-EPI-based DTI-derived metrics (ICCs ranged from 0.843 to 0.914). TAO group showed significantly lower FA (*P* < 0.001) and higher ADC (*P* = 0.014) of LG than HC group. Active subgroup had significantly lower FA (*P* < 0.001) and higher ADC (*P* < 0.001) of LG than inactive subgroup (Table 2; Fig. 2).

**Table. 2:** rs-EPI-based DTI parameters of lacrimal gland between groups

	**TAO**			**HC**	^***a***^***P ***
	**Active**	**Inactive**	^***b***^***P***		
**FA**	0.313±0.067			0.375±0.082	<0.001
	0.277±0.040	0.349±0.071	<0.001		
**ADC**	1.403±0.276			1.289±0.172	0.014
	1.477±0.342	1.329±0.164	<0.001		

^a^*P* Comparisons between TAO and HC

^b^*P* Comparisons between active phase and inactive phase

The unit of ADC is ×10^-3^ mm^2^/s

*rs-EPI-based DTI* readout-segmented echo-planar imaging-based diffusion tensor imaging, *FA* fractional anisotropy, *ADC* apparent diffusion coefficient

**Fig. 3:**
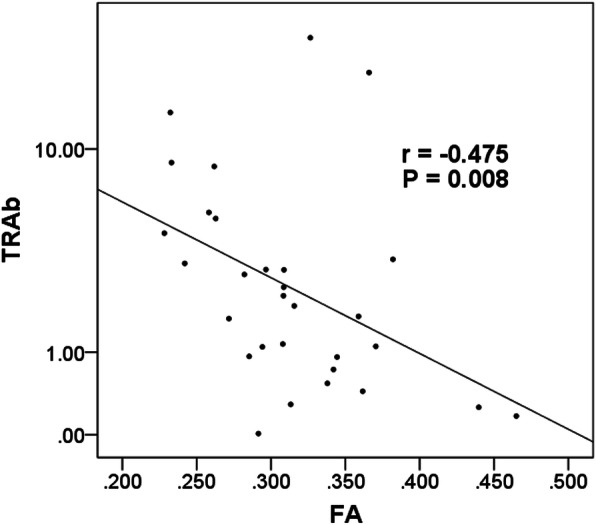
Scatter diagrams showing the correlations between FA of LG and serum level of TRAb. The unit of TRAb is IU/L. TRAb: thyrotrophin receptor antibody

In TAO group, FA of LG was negatively correlated with TRAb (*r*=-0.475, *P* = 0.008) (Fig. 3), whilst ADC of LG showed no significant correlation with any of the serum thyroid biochemical markers (*P* > 0.05).

**Fig. 4:**
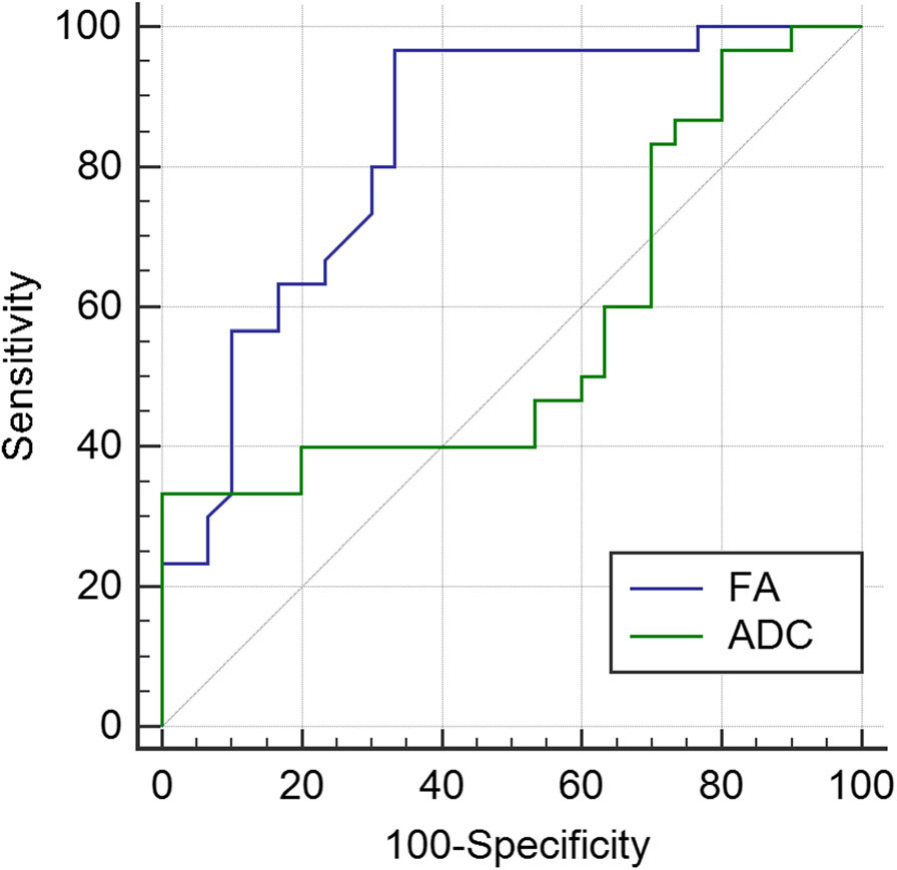
Receiver operating characteristic curves of using FA and ADC of LG for grading disease activity

For discriminating disease activity, FA value showed significantly higher area under curve, compared with ADC (0.832 vs. 0.570, *P* = 0.009) (Fig. 4). A cut-off FA value of 0.322 for diagnosis of active TAO was associated with a sensitivity of 96.7 % and a specificity of 66.7 %. Similarly, a cut-off ADC value of 1.604 was associated with a sensitivity of 33.3 % and a specificity of 100.0 %.

## Discussion

Our study dedicated in the potential of rs-EPI-based DTI in characterizing the pathological alterations of LG, and discriminating the disease activity in TAO patients. Three main findings were discovered. First, TAO patients had significantly lower FA and higher ADC of LG compared with healthy subjects. Meanwhile, active TAOs shared significantly lower FA and higher ADC compared with inactive ones. Second, FA value of LG showed significantly negative correlation with TRAb, whereas ADC did not. Third, FA value of LG demonstrated a more desirable discriminating performance for disease activity than ADC.

In this work, ADC value of LG differed significantly between TAO and HC groups, as well as between active and inactive subgroups. This finding was in accord with previous study of Razek et al. which used diffusion-weighted imaging to assess the changes of LG [27]. Similar to extraocular muscles, LG was thought to experience an initially acute inflammation characterized by mononuclear cell infiltration, fibroblast proliferation and interstitial edema, and subsequently chronic inactive plateau featured with interstitial fibrosis, and fatty infiltration [26, 27]. Considering that diffusivity value, reflecting the magnitude of water diffusion dominated by interstitial space [28], could increase in edematous tissue and decrease in fibrotic and fatty infiltrated tissue [26, 29], it is reasonable that the LG of TAO patient, especially that of the active one would present elevated ADC value.

FA represents the directionality of diffusion of water molecules and could reflect integrity of microstructural architectures [15–17]. As a novel finding of this study, TAO patients were detected to have decreased FA values of LG than healthy subjects. Prior studies focused on nervous system and muscles usually attributed reduction of FA to fiber destruction or displacement [13, 14, 29]. Analogously, we hypothesized that the lower FA of the patient group might also be associated with the disorganization, cell lysis, or acinar disruption of involved LG tissue. Further subgrouping analysis exhibited that active TAOs had decreased FA in contrast to inactive mimics. Previously, it has been suggested that FA could be largely affected by cell density [30]. Malignant tumors with higher cellularity have been documented to show higher FA values than benign mimics in various organs, supposed to result from the relatively limited extracellular space and subsequently increased directionality of water diffusion [21, 30, 31]. Therefore, in view of the primary histological change of interstitial edema within LG tissue in active phase, which could lead to increased extracellular space together with probably decreased water directionality, it could be natural that active TAOs would show lower FA values than inactive patients.

In the present study, FA value of LG displayed significant correlation with serum level of TRAb, while ADC did not. Furthermore, FA outperformed ADC in discriminating disease activity. According to our database, FA seemed to demonstrate more potency in representing lacrimal dysfunction and disease process than ADC. Similar superiority of anisotropy to diffusivity have also been reported in previous studies regarding the discriminations between benign and malignant lesions on parotid gland and breast [15, 21]. In our opinion, although several factors, such as extracellular matrix, extracellular-to-intracellular space ratio and tortuosity, could affect FA and ADC simultaneously [32], the alterations of these two metrics under certain microenvironment might still be discrepant. It depends on the different weights and complex interactions of all factors. Nonetheless, based on our results, we insisted that the DTI-derived metrics, particularly the FA value, might be a promising imaging marker for the non-invasive assessment of LG. It may assist in discriminating the disease activity, and subsequently help the clinicians to make accurate treatment strategy.

Our study has a few limitations. First, the study cohort was relatively small. Second, due to the retrospective nature, synchronous biochemical information of tear or histological analysis of LG was difficult to be available. Further larger sample study with lacrimal biochemical examinations would be needed to verify our findings and clarify the exact pathological mechanism.

## Conclusions

Our preliminary study indicated that rs-EPI-based DTI is able to detect microstructural alterations of LG in patients with TAO. The derived metrics, particularly FA, hold the potency to reveal disease activity.

## Data Availability

The datasets generated during and analyzed during the current study are not publicly available due to patient protections and institutional policy but are available from the corresponding author on reasonable request.

## Abbreviations

ADC: Apparent diffusion coefficient
CAS: Clinical activity score
DTI: Diffusion tensor imaging
FA: Fractional anisotropy
FT3: Free triiodothyronine
FT4: Free thyroxine
HC: Healthy control
ICC: Intraclass correlation coefficient
LG: Lacrimal gland
MRI: Magnetic resonance imaging
rs-EPI: Readout-segmented echo-planar imaging
ss-EPI: Single-shot echo-planar imaging
TAO: Thyroid-associated ophthalmopathy
TRAb: Thyrotrophin receptor antibody
TSH: Thyroid-stimulating hormone
